# Strain induced electronic structure variation in methyl-ammonium lead iodide perovskite

**DOI:** 10.1038/s41598-018-25772-3

**Published:** 2018-05-17

**Authors:** Le Zhang, Wei Geng, Chuan-jia Tong, Xueguang Chen, Tengfei Cao, Mingyang Chen

**Affiliations:** 1 Beijing Computational Science Research Center, Beijing, 100193 China; 2 School of Materials Science and Engineering, Sun Yat-sen University, Guangzhou, 510275 P. R. China; 3 School of Material Science and Engineering, Hebei University of Technology, Tianjin, 300130 P. R. China

## Abstract

Methyl-ammonium lead iodide perovskite (CH_3_NH_3_PbI_3_) has drawn great attention due to its excellent photovoltaic properties. Because of its loosely compacted structure, the structural, electronic and optical properties of CH_3_NH_3_PbI_3_ are sensitive to external modulations. Strain effects on CH_3_NH_3_PbI_3_ are fully investigated by the first principles calculations. The results indicate that the inorganic framework deforms under compression or stretch and the embedded organic CH_3_NH_3_+ molecules rotate correspondingly. A band gap oscillation and a new structural phase in response to the external strain were observed for the first time. These phenomena are explained with the nonlinear structural deformation and phase transition under the external strains. The semi-quantitative relationship between the band gap variation and geometry change under the external strain is obtained. We found that the shift of valence band maximum under the external strain is mostly determined by the most stretched or compressed Pb-I bond of CH_3_NH_3_PbI_3_, and the shift of the conduction band minimum under the external strain is likely to be determined by the largest Pb-I-Pb bond angle in the system. These results are important for understanding of strain effects on semiconductors and guiding the experiments to improve the performance of the perovskite solar cells.

## Introduction

Methyl-ammonium lead iodide perovskite (CH_3_NH_3_PbI_3_) has become a stellar photovoltaic material due to its suitable band gap, wide absorption range for visible light, and low manufacturing cost^1–6^. Early in 2009, Miyasaka *et al*. firstly investigated the CH_3_NH_3_PbI_3_-based liquid dye-sensitized solar cells (DSCs) and the power conversion efficient (PCE) is about 3.8%^1,2^. Since then, extensive experimental efforts have been focused on increasing the PCE of perovskite-based solar cells. A variety of methods have been proposed, such as varying the photoelectrode materials^3–6^, halogens doping^1,7,8^, varying the organic cations, and adopting the mesoporous semiconductor layers^6,9–11^. So far, PCE over 20% has been achieved with the hybrid halide perovskite^12,13^. Furthermore, plenty of theoretic works have been carried out to investigate the fundamental mechanisms of photovoltaic properties of this class of materials, and to predict characteristics beyond experimental observations^14,15^, such as spontaneous polarization, possible ferroelectricity property, and potential practices to enhance the photovoltaic properties^16–19^. It has been indicated that these properties are closed related to the ground state electronic structures. Several strategies including doping^20^, morphology controlling^11,21^, halogen elements mixing^22–24^, and strain engineering have been put forward to tune the electronic structure of perovskite materials^25–27^. Among them, strain modulation is the most commonly used technique, because of its efficient modulation effects and relatively easy realizations. Strain modulation can affect the atomistic structure of the material which in turn reshape the electronic structure.

In the electronic structure calculations, strain can be introduced by the external stress field, the structural deformation or the interface stress. The structural deformation can be caused by ion dopants that induce steric effects. In 2008, Borriello *et al*. found that lattice size, structure stability and band gap of bulk perovskites were sensitive to the choices of organic cations^16,28,29^. Although the cation of the perovskite does not directly contribute to the valence band edge (dominated by p orbitals of I) or conduction band edge (dominated by p orbitals of Pb), the substitution of inorganic Cs in CsPbI3 with organic cations CH_3_NH_3_+ or CH(NH_2_)_2_+ shifts the energy levels of Pb-I anti-bonding orbitals at the valence band edge and bonding orbitals at the conduction band edge mainly by altering the Pb-I-Pb angles in the material, and can hence efficiently tailor the electronic structure of perovskite^30,31^. Moreover, in actual devices, perovskite solar cell has a sandwich-like structure, where the absorption layer, i.e. CH_3_NH_3_PbI_3_ perovskite, is placed between the hole (or electron) transport layer and the window layer. Thus, lattice mismatches occur inevitably at the interface^16,32^, which also introduce strains to the perovskite layer that may affect the PCE of perovskite photovoltaic devices. However, there are hitherto very few systematic theoretical explorations about the strain effects on electronic structure and photovoltaic property of CH_3_NH_3_PbI_3_, which trails rapidly growing experimental discoveries.

Here, we perform systematic simulations to investigate the strain effects on bulk CH_3_NH_3_PbI_3_. The deformation of the material, orientations of the organic molecules, and electronic structure and optical adsorption properties of the material under different compressions and tensile strains are carefully analyzed. Our results reveal that the external strains greatly affect orbital hybridization of inorganic skeleton as well as the orientations of the embedded molecules. The electronic structures as well as the optical properties are changed correspondingly. In particular, the band gap of CH_3_NH_3_PbI_3_ exhibits an abnormal oscillation, which can be explained by structural distortion, orbital hybridization as well as orientations of the organic cations.

## Results and Discussion

**Figure 1:**
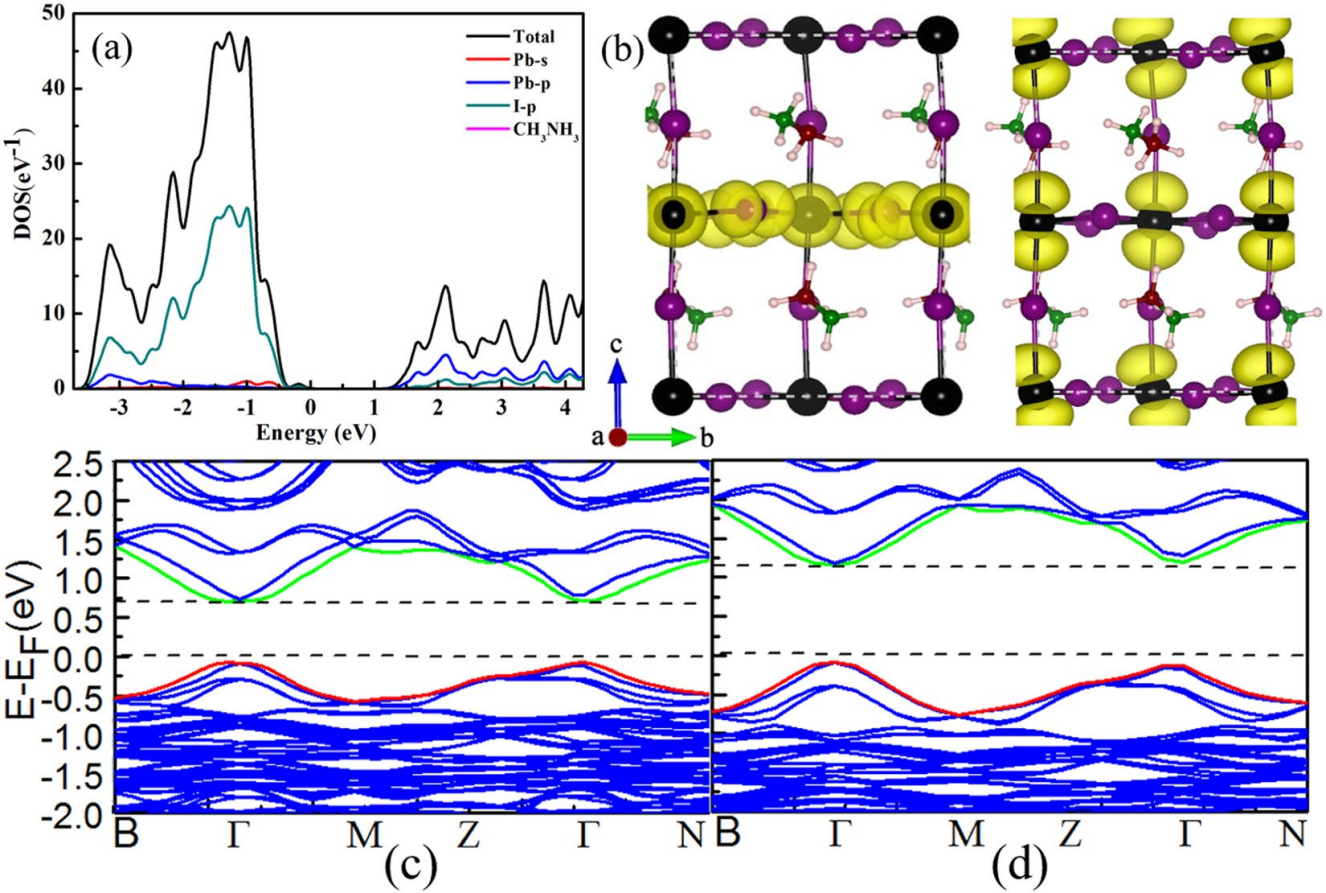
The projective density of states (PDOS) of tetragonal phase CH_3_NH_3_PbI_3_ (**a**), and charge density distribution of valence band maximum (VBM) (middle column), and conduction band minimum (CBM) (right column) (**b**). The energy band of CH_3_NH_3_PbI_3_ with PBE plus SOC (**c**) and HSE plus SOC calculations (**d**). The CBM was set to zero in PDOS. The black, purple, brown, green, and pink balls are lead, iodine, carbon, nitrogen, and hydrogen atoms, respectively. The yellow part represents the charge distribution.

As shown in Fig. 1(b), the CH_3_NH_3_PbI_3_ is composed by periodic cages of which each vertex is a PbI_6_ octahedron, filled by CH_3_NH_3_^+^ cations. The supercell we used for the CH_3_NH_3_PbI_3_ simulation system consists of two layers of CH_3_NH_3_^+^ cations. CH_3_NH_3_^+^ molecules of the same layer have the same orientations. The orientations of CH_3_NH_3_^+^ molecules of the upper layer are opposite to those of the bottom layer. The calculated band gap for CH_3_NH_3_PbI_3_ is 1.45 eV at the PBE level with D3 van der Waals corrections (PBE + vdW-D3), which is 0.18 eV and 0.10 eV smaller than that of previous theoretical prediction of 1.63 eV (PBE + vdW-DF) and experimental value of 1.55 eV^33^. The band gap for CH_3_NH_3_PbI_3_ was also predicted using the PBE and hybrid Heyd-Scuseria-Ernzerhof (HSE06) functionals with the inclusion of the spin-orbit coupling (SOC) effect (Fig. 1(c,d) respectively). The corresponding band gaps are determined to be 0.75 eV (Fig. 1(c)) and 1.20 eV (Fig. 1(d)), respectively, which are 0.70 eV and 0.25 eV smaller than the experimental value. Therefore, the band gap predicted using the PBE functional without SOC agrees best with the experimental measurements and is likely to be reliable to descript the electronic structure of CH_3_NH_3_PbI_3_.

The projected density of states (PDOS) and charge density distribution of the perfect CH_3_NH_3_PbI_3_ were also calculated and corresponding results are shown in Fig. 1(a,b), respectively. It can be observed from PDOS that the conduction band minimum (CBM) is dominated by the p orbitals of Pb atoms and the valence band maximum (VBM) is mainly composed by the p orbitals of I atoms. Being consistent with PDOS, the partial charge distribution also indicates that the electron density related to the VBM is mainly located on the I atoms and thus implies the Pb-I ionic anti-bonding orbitals, whereas the electron density of the CBM is mainly found on the Pb atoms and indicates the Pb-I ionic anti-bonding orbitals. It is noted that the CBM extends over the whole structure, while, on the other hand, the VBM is only located at the middle layers of the bulk PbI_6_ skeleton.

**Figure 2:**
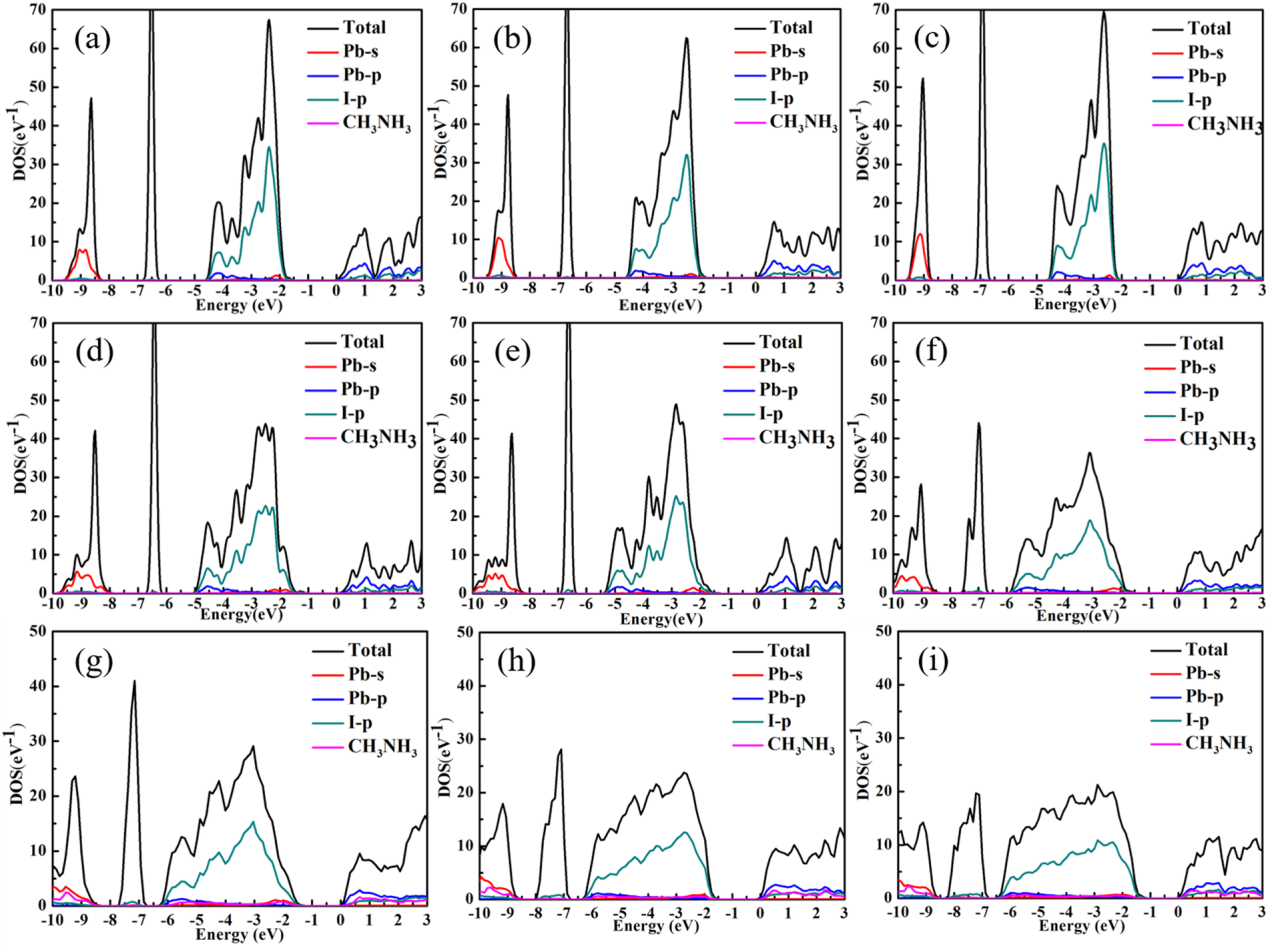
PDOS of CH_3_NH_3_PbI_3_ under 2.5% (**a**), 5% (**b**), 7.5% (**c**), −2.5% (**d**), −5% (**e**), −7.5% (**f**), −10% (**g**), −12.5% (**h**), −15% (**i**) strains. The energy level for CBM is set to zero.

**Figure 3:**
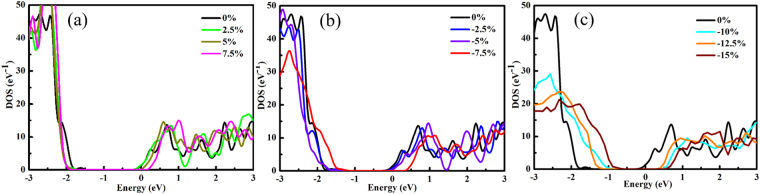
Aligned TDOS for the CH_3_NH_3_PbI_3_ systems under various strains: 2.5% to 7.5% (**a**), −2.5% to −7.5% (**b**), and −10% to −15% (**c**). I core s band is used as the reference for the alignment.

To investigate strain effects on CH_3_NH_3_PbI_3_, the PDOS (Fig. 2) of CH_3_NH_3_PbI_3_ under different strains (under up to −15% compression strain and up to 7.5 tensile strain) are examined. The lattice parameters for the system under each tested external strain were tabulated in Table S1 in the supplementary information. The PDOS diagrams show that the major components of the VBM and CBM are not changed under different strains. As the tensile strain increases, the valence band gradually narrows and becomes dense. In contrast, under compression strains the valence band broadens. To further understand the correlations between the applied strain and the resulting of the electronic structure. We aligned the total DOS for the CH_3_NH_3_PbI_3_ systems under various strains (Fig. 3 and enlarged drawing in Fig. S1). The alignment was done by choosing the deep, sharp band arising from the I core s orbital as the reference. The tensile strains are found to shift the VBM to lower energy and shift the CBM to the higher energy simultaneously (Fig. 3(a)), resulting in a net increase in the band gap. The shift of VBM under the tensile strains is insensitive to the magnitude of the applied stress, whereas the shift of the CBM under the tensile strains increases linearly as the tensile strain increases. This is consistent with the predicted linear response of the band gap of CH_3_NH_3_PbI_3_ to the external tensile strain. The −2.5% and −5% compression strains shift the VBM and CBM to higher energy (Fig. 3(b)). Under the −2.5% strain, the shift of the VBM (~0.17 eV) is greater than the shift of the CBM (~0.03 eV), which resulting in the slight decrease in band gap as compared to the original structure. Increasing the compression strain to −5%, the VBM shift about ~0.06 eV and increases the CBM shift about ~0.12 eV. The opposite responses of the electronic structures of CH_3_NH_3_PbI_3_ under −2.5% and −5% strains are due to the phase transition from the tetragonal phase to orthorhombic phase induced by the compression strain, and more discussion can be found below. Further increasing the compression strain will shift the VBM and CBM to even higher energy (Fig. 3(b,c)). The net effect of shifting both the VBM and CBM under large compression strain causes the slightly increase in the band gap as compared to the band gap of CH_3_NH_3_PbI_3_ under −5% compression strain. It is also noted that the valence band at ~−3 eV narrows under the influences of the tensile strains (Figs 2 and 3), and the center of this valence band is slightly shifted to the higher energy despite the VBM is shifted to the lower energy; the conduction band essentially maintains original shape and are slightly shifted to the higher energy. Therefore, we expect that the low energy optical absorption (in the red and infrared regions) will not be significantly change under the small tensile strains (≤2.5%), and the high energy absorption (in the blue regions) will be slightly reduced due to the valence band narrowing. Both the low energy and high energy absorptions are expected to be suppressed by the moderate tensile strains due to the substantial increase in the band gap as well as the valence band narrowing. On the other hand, small compression strains ≤ |−2.5%| does not have substantial impacts on the band gap, the shape of the valence band, or the shape of the conduction band, and thus will only have small impact on the optical absorption property. The moderate and large compression strains increase the band gap by a significant amount and also broaden the valence band and conduction band. As a consequence, the low energy optical absorption is expected to be suppressed and the high energy optical absorption is expected to be enhanced for CH_3_NH_3_PbI_3_ under the moderate and large compression strains.

**Figure 4:**
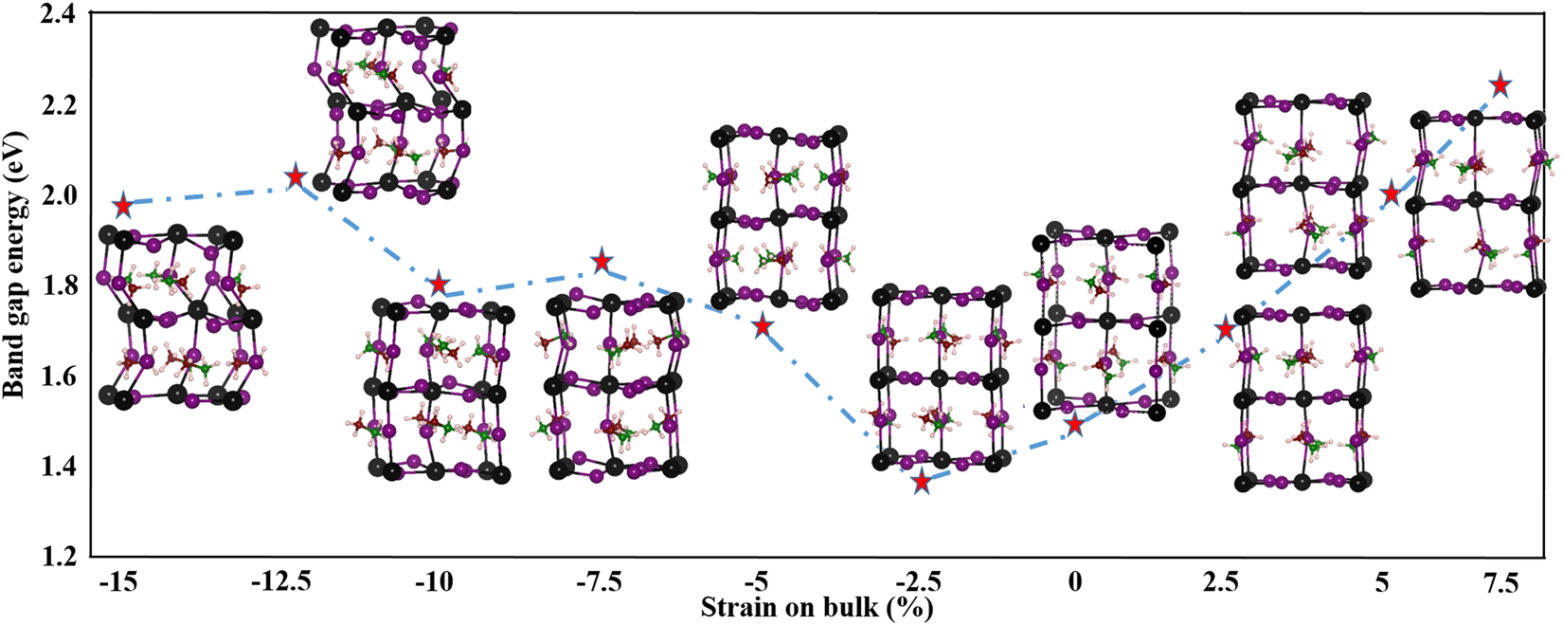
The band gaps (red stars) and corresponding structures of CH_3_NH_3_PbI_3_ under different strains (denote as ε). We abbreviation the curve as E_g_–ε.

Based on the PDOS results, the band gaps of CH_3_NH_3_PbI_3_ systems under different strains are summarized and shown in Fig. 4. As shown in Fig. 4, the band gap is dependent on the magnitude and direction of the applied strain. From −2.5% compression to 7.5% tensile strain, the band gap is approximately a linear function of the applied strain. The band gap value essentially increases as compression strain increases (*i.e*. strain becomes more negative) from −2.5% and −15.0%, and small oscillation of the band gap-strain curve is observed. The non-linear response of the band gap of CH_3_NH_3_PbI_3_ to the external compression strains can be ascribed to the non-linear structural deformation under large compression strains. The geometry configurations of CH_3_NH_3_PbI_3_ under different strains were shown in Figs 4 and S2.

The structures under tensile strains have the same tetragonal phase as the original structure. Even though the small and moderate tensile strains do not induce phase transition of CH_3_NH_3_PbI_3_, they affect the geometry of CH_3_NH_3_PbI_3_ substantially indicated by the changes in the bond lengths and bond angles. Each Pb atom of CH_3_NH_3_PbI_3_ is coordinated to 6 I atoms, forming three comparatively long Pb-I bonds and three short Pb-I bonds. At zero external strain, the long Pb-I bonds are only slightly longer (<0.15 Å) than the shorter bonds. Increasing the tensile strain enlarge such differences, and hence reduces the local point group symmetry of the Pb centers (pseudo-octahedral symmetry under 0% strain). Under the 7.5% tensile strain, the longest Pb-I bond is about 1 Å longer than the shortest Pb-I bond in the system. The local point group symmetry reduction of the Pb centers is also indicated by the scattering of the Pb-I-Pb bond angles at tensile strains. For example, one of the two Pb-I-Pb bonds along c-axis about a Pb center becomes more bent as the tensile strain increases.

Under the −2.5% compression strain, the CH_3_NH_3_PbI_3_ has the same tetragonal phase as the unstressed structure. Increasing the compression strain to −5%, the geometry optimization of the system leads to an orthorhombic phase. In the orthorhombic phase, the I atoms on the Pb layers (on the ab-plane) are vertically aligned and the I atoms between the Pb layers are misaligned, which is opposite to I alignment patterns for the tetragonal phase under zero external pressure^34^. Further increasing the compressing strain (≥|−7.5%|) causes another phase transition from the orthorhombic phase to the distorted tetragonal phase. The distortion can be indicated by the misalignment of the inter-layer I atoms. In contrast to the systems under the tensile strains, the small and moderate compression strains (<|−7.5%|) tend to increase the local point group symmetry of the Pb centers, which can be implied by the more comparable Pb-I bond distances about a Pb center. This is because at low compression strains, the geometry become more condensed and more uniform. However, the large compression strains (≥|7.5%|) tend to reduce the local point group symmetry of the Pb coordination shells when the geometry becomes distorted. The distortion is due to the prominent steric effect of CH_3_NH_3_^+^ under large compression strains.

The geometry visualizations for CH_3_NH_3_PbI_3_ under various strains along the a and c axes are shown in Fig. S2 in the supplemental information. The CH_3_NH_3_PbI_3_ under the 2.5–7.5% tensile strains and under the −2.5% compression strains are confirmed to have the same tetragonal phase as the unstressed structure. The −5% compression strain turns the structure into the orthorhombic phase. Under the −7.5% compression strain, the phase of the structure looks similar to the tetragonal phase along the c axis but has a different view than the tetragonal phase along the a and b axes. The PbI_6_ fragments of the CH_3_NH_3_PbI_3_ remains regular octahedral but appear misaligned in all three directions. Increasing the compression strain to −10%, the structure exhibits a phase similar to the tetragonal phase. PbI_6_ fragments are more distorted than the fragment in the structures under smaller compression strains, the misalignments along all three directions are significant smaller than those in the structure under −7.5% compression strain, indicating a minor phase transition at the −10% strain. Under the −12.5% and −15% strains, not only do the PbI_6_ fragments of CH_3_NH_3_PbI_3_ become more deformed, octahedral along the c direction form zigzags. The distorted structures under the −12.5% and −15% strains are alike ascribed to a new phase. Overall, under for strains ranging from −15% to 7.5%, the CH_3_NH_3_PbI_3_ mainly exhibits four different phases, including the tetragonal phase (−10% and −2.5 to 7.5%), the orthorhombic phase (−5%), the asymmetric phase (−7.5%) and the zigzag phase (−12.5% and −15%). The asymmetric phase and the zigzag phase for CH_3_NH_3_PbI_3_ were identified for the first time.

It is noted that not only do the Pb-I bond lengths and Pb-I-Pb angles change with the external strain, but the orientations of CH_3_NH_3_^+^ molecules also change under the external compression strain. The orientations and alignment pattern of the CH_3_NH_3_^+^ molecules are indicators for geometry changes of the periodic PbI_6_^−^ cage under the external strains. In the structures under small and moderate strains (tensile or compression), namely the tetragonal, the orthorhombic and the asymmetric phases, the nearest CH_3_NH_3_^+^ pairs across two different layers of CH_3_NH_3_PbI_3_ are found to be parallel to each other in opposite orientations. In the zigzag-phase structure formed under large compression strains, there exists two types of interlayer CH_3_NH_3_^+^ pairs, a parallel pair and a perpendicular pair. In addition, under the large compression strain, the distances between the CH_3_NH_3_^+^ molecules and the cage become smaller; the periodic cage formed by PbI_6_^−^ tends to wrap around the CH_3_NH_3_^+^ molecules, and thus become severely distorted. Consequently, the CH_3_NH_3_^+^ molecules start to contribute to the valence band and conduction band, although the contribution is fairly small (Fig. 2). The influence of CH_3_NH_3_^+^ in determining the band gap of CH_3_NH_3_PbI_3_ is indirect yet is critical. The geometry changes under external strains are greatly molded by the CH_3_NH_3_^+^ via steric and electronic effects, which becomes more apparent in the structures under large external strains.

**Figure 5:**
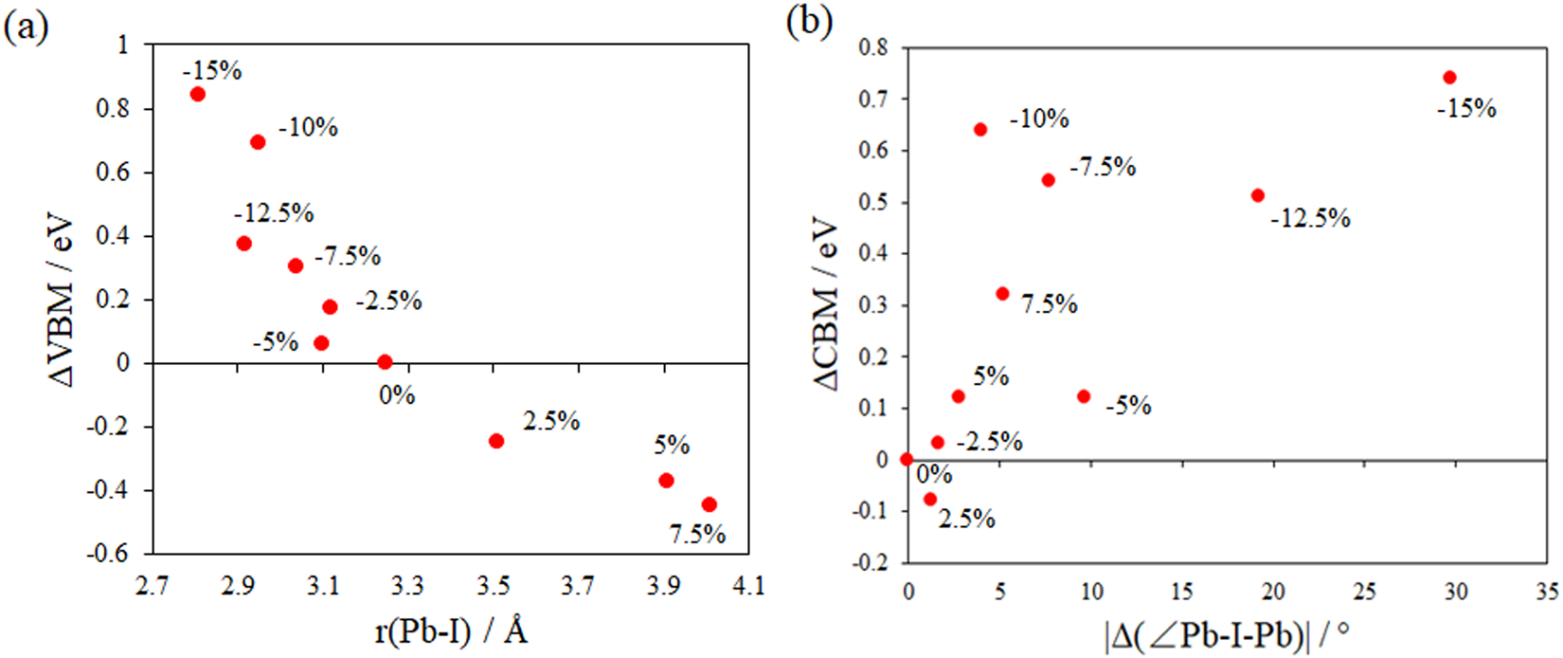
(**a**) Relationship between the VBM shifts ΔVBM and the “least stable” Pb-I bond of CH_3_NH_3_PbI_3_ under various external strains. (**b**) Relationship between the CBM shifts ΔCBM and |Δ(∠Pb-I-Pb)| of CH_3_NH_3_PbI_3_ under various external strains. ΔVBM and ΔCBM are the difference between the VBM/CBM of the stressed CH_3_NH_3_PbI_3_ and unstressed CH_3_NH_3_PbI_3_. |Δ(∠Pb-I-Pb)| is the absolute of difference between the largest ∠Pb-I-Pb angles of the stressed and unstressed CH_3_NH_3_PbI_3_ systems.

The response of the band gap under the external strain can be correlated to the geometry changes (Table 1). The valence band edge is mainly attributed by the Pb-I anti-bonding orbitals that is dominated by the I p orbitals. Therefore, the VBM of CH_3_NH_3_PbI_3_ is contributed by the highest energy Pb-I anti-bonding orbitals that involves the least stable I p electron. Approximately, the I atom that forms the Pb-I bond with bond length differs most from the Pb-I bond distance in the unstressed system (~3.25 Å) is considered as the “least stable” I atom of a CH_3_NH_3_PbI_3_ system, and the corresponding Pb-I bond is considered as the “least stable” Pb-I bond. The VBM shifts under the external strains with reference to the unstressed system obtained from the aligned total DOS diagrams are plotted against the bond length of “least stable” Pb-I bond (Fig. 5(a)). A qualitatively linear correlation is found between the VBM shift and the “least stable” Pb-I distance. This implies that the most stretched or compressed Pb-I bond in the system determines the VBM shift under the external strain therein. Similarly, the CBM of CH_3_NH_3_PbI_3_ is contributed by the lowest energy Pb-I anti-bonding orbital, which appears as a valence hole of a Pb atom. The “most stable” Pb-I anti-bonding orbital, however, is much harder to determine. This is not only because Pb is coordinate to six I atoms, but also because the crystal field around Pb depends on multiple factors including the Pb-I-Pb angles. We are only able to show there is likely an approximate linear correlation between the CBM shift and the |Δ(∠Pb-I-Pb)| of CH_3_NH_3_PbI_3_ under various external strains, where |Δ(∠Pb-I-Pb)| is the absolute of difference between the largest ∠Pb-I-Pb angles of the stressed and unstressed CH_3_NH_3_PbI_3_ systems. We tentatively suggest that the VBM response under external strain is mostly determined by the largest ∠Pb-I-Pb angle in the stressed system.

**Table 1:** Optimized geometry parameters under different strains.

Strain (%)	Pb-I-Pb bond angles (°)		Pb-I bond length (Å)	
	In ab plane	Along c axis	In ab plane	Along c axis
7.5	144.0–176.6	154.7–170.7	3.02–4.01	3.10, 3.89
5	141.2–172.6	159.3–173.1	3.01–3.91	3.16, 3.80
2.5	148.8–160.4	167.5–174.6	3.13–3.51	3.19, 3.50
0	147.8–165.7	174.1–175.9	3.17–3.31	3.20, 3.30
−2.5	146.7–159.3	176.5–177.6	3.13–3.21	3.12, 3.17
−5	145.6–146.3	165.8–166.2	3.14–3.15	3.10, 3.11
−7.5	132.2–163.4	139.9–168.2	3.06–3.10	3.04, 3.14
−10	122.1–164.6	147.3–171.9	2.99–3.10	2.95, 3.07
−12.5	110.9–155.1	126.6–156.7	2.92–3.08	3.02, 3.23
−15	124.3–156.6	125.7–146.2	2.81–3.09	2.96, 3.24

**Figure 6:**
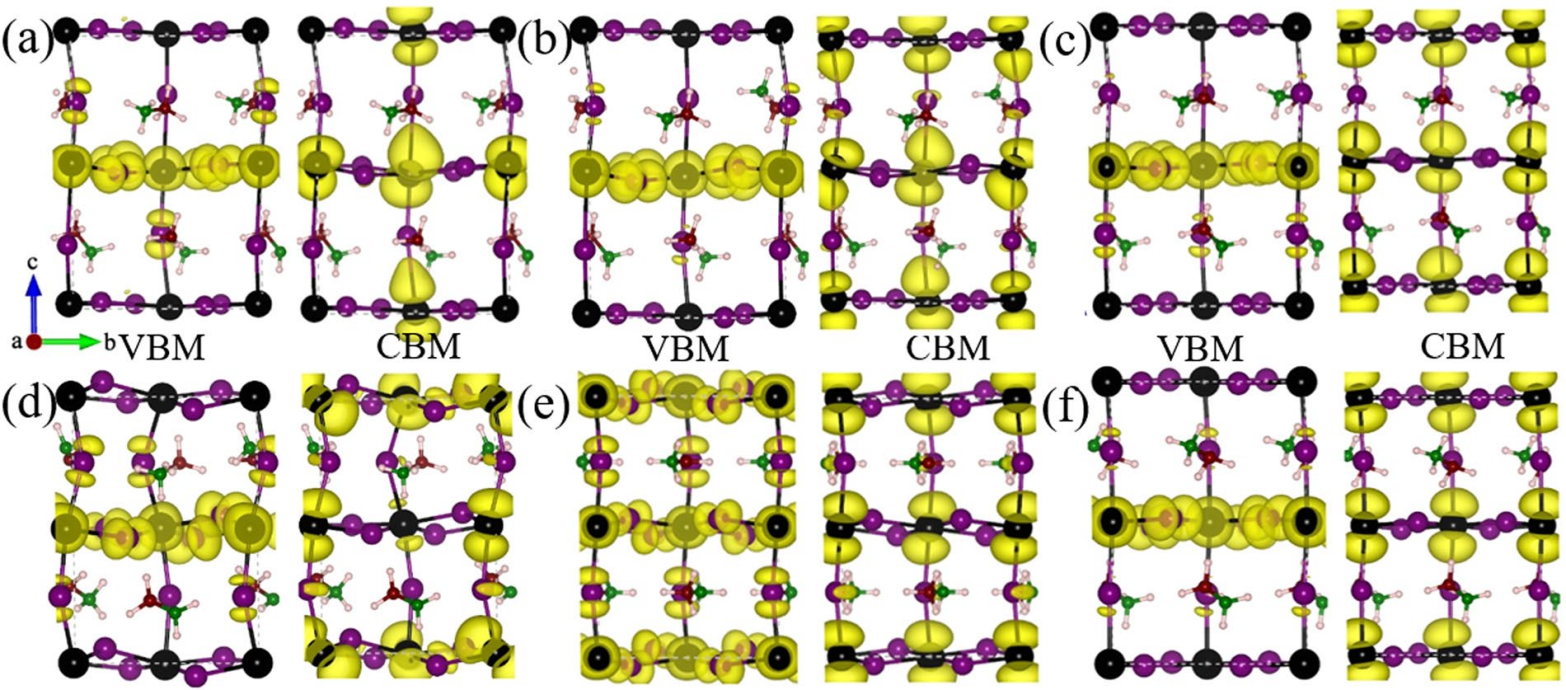
Calculated charge density distribution of VBM and CBM under 2.5% (**a**), 5% (**b**), 7.5% (**c**), −2.5% (**d**), −5% (**e**) and −7.5% (**f**) strains, respectively.

The partial charge density distributions under different external strains are also examined. As shown in Fig. 6, the spatial distributions at the VBM and CBM are alike for structures under 2.5% and 5% tensile strains, as for both cases the strain is small and the geometry is close to the original bulk geometry. Under 7.5% tensile strain, the CBM is no longer spatially attributed over all the Pb atoms. Such a change is due to the reducing of symmetry with increased strains caused by the increased bond distance difference between the long and short Pb-I bonds. The degeneracy of the conduction band edge is therefore changed, and the VBM electron becomes more localized. For structures under −2.5% and −7.5% compression strains, the charge density of VBM and CBM are similar, although the charge densities of the structure under −7.5% appear to be more deformed due to the geometry distortion. This is because both structures have the same tetrahedral phase. The structure under −5% compression strain exhibits a totally different VBM density that other structure under compression strains. The VBM density is delocalized and spreads over all I atoms, as the structure under −5% compression strain is in an orthorhombic structural phase that is in higher symmetry than the unstressed bulk structure (in the tetrahedral phase).

**Figure 7:**
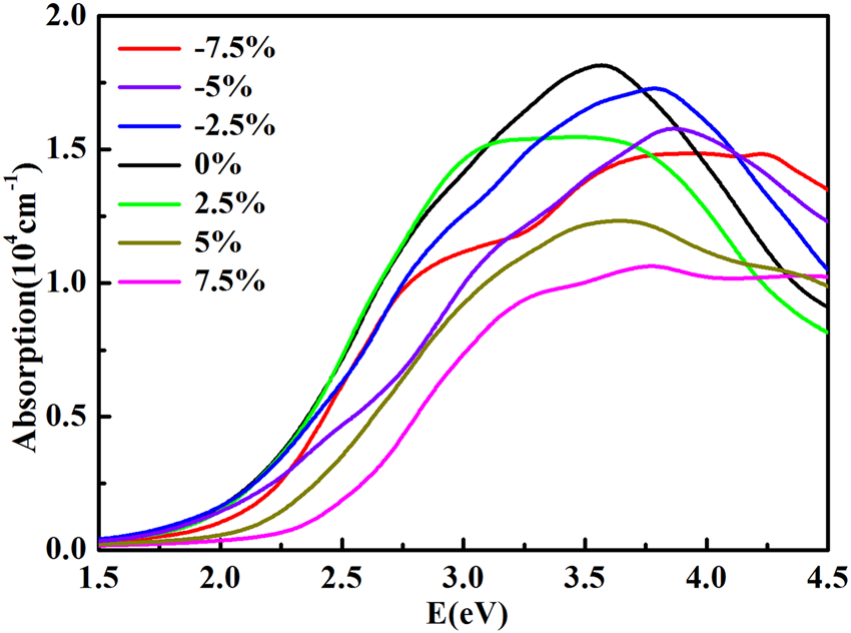
Calculated absorption coefficients of CH_3_NH_3_PbI_3_ under different strains.

The light absorption properties of photovoltaic material are greatly impacted by their electronic structures. The strain induced band gap variation of CH_3_NH_3_PbI_3_ implies that its optical adsorption properties are also modulated by external strains. As shown in Fig. 7, under both tensile or compression strains, the absorption intensity of CH_3_NH_3_PbI_3_ for incident light energy window of 2.5 eV to 3.5 eV is slightly reduced. Except for the absorption curve for the structure under the 2.5% tensile strain, all the other curves of the strained structures show blue shifts. The structures under strains have lower absorption strength at low energy but are found to have better absorption properties than the unstressed bulk structure at energy greater than 4 eV. Which is consistent with the predicted electron structures. Substantial blue shifts are found for structures under large strains, implying such strains deform the crystal structure of CH_3_NH_3_PbI_3_ and undermines its photovoltaic properties.

## Conclusions

In summary, the strain effects on the structures, electronic properties and optical properties of CH_3_NH_3_PbI_3_ are systematically studied. Our results show that, because of its loosely compacted structure, the property of CH_3_NH_3_PbI_3_ can be readily changed by the external strains. The electronic properties and optical properties can be effectively alternated by modulating the material with external strains. It is interesting that the band gap of CH_3_NH_3_PbI_3_ oscillates as the applied compression strains monotonically increases, which is totally different from the behaviors of the conversional semiconductors. Such abnormal variations can be explained with the nonlinear structural deformations and phase transitions under large external strains. The semi-quantitative relationships between the VBM/CBM and the geometry under the external strains were extracted. This work reveals an easy way to tailor electronic structures of CH_3_NH_3_PbI_3_, and also contributes to basic understandings to perovskite photovoltaic materials.

## Computational Details

All the calculations were performed with the Vienna Ab initio Simulation Package (VASP)^24,35^, and the Perdew-Burke-Ernzerhof (PBE) formulation of the generalized gradient approximation (GGA) was used to describe the exchange-correlation interaction^36,37^. The projector augmented-wave method (PAW)^38^ was employed to treat valence-core interactions. The van der Waals (vdW) correction was considered with the Grimme approach (DFT-D3)^39^. The energy cutoff for the basis set was set to 500 eV. The Brillouin zone was sampled with a 4 × 4 × 4 k-mesh of Monkhorst-Park scheme^40^. All structures were fully relaxed until the residual force on each atom is less than 0.01 eV/Å.

In this study, tetragonal phase of CH_3_NH_3_PbI_3_ was first analyzed because of its room temperature stability, and excellent light adsorption property in visible light regions. The supercell is constructed using 1 unit cell, which contains 24 atoms with a chemical formula of CH_3_NH_3_PbI_3_. For the structure under zero external strain, the lattices are fully relaxed and the resulting lattice constants are 8.94 Å, 8.94 Å and 12.98 Å, which are comparable with the experimental values (8.87 Å, 8.87 Å, 12.67 Å)^34^. To simulate the strain effects on CH_3_NH_3_PbI_3_, we used the volume strain method for the simulation by applying the certain percentage strains along three axes. The three lattice lengths, a, b and c, were scaled equally by a percentage and then fixed, while the lattice angles were fixed as in the unstressed crystal structure. The atoms in the supercell were then fully relaxed to obtain the optimized structure for CH_3_NH_3_PbI_3_ under the corresponding strain. To investigate the general trends of the varying electronic and optical properties under strains, the bulk structure was calculated under strains (in terms of the change of the lattice constants with respect to the original crystal structure under no strains) ranging from −15.0% to 7.5% with a 2.5% interval. Here, the negative (positive) values represent the uniformly compression (tensile) strains on bulk CH_3_NH_3_PbI_3_.

## Electronic supplementary material

Supplementary Information
