# Supplementary material for: Strain induced electronic structure variation in methyl-ammonium lead iodide perovskite
Source: Sci Rep. 2018 May 17;8:7760. doi: 10.1038/s41598-018-25772-3 (PMC5958122; doi:10.1038/s41598-018-25772-3)
Supplement: Supplementary file 1 — Supplementary Information [file 41598_2018_25772_MOESM1_ESM.docx]

**Supplementary Information**

**Strain induced electronic structure variation in methyl-ammonium lead iodide perovskite**

*Le Zhang^1^, Wei Geng^2^, Chuan-jia Tong^1^, Xueguang Chen^3^*, Tengfei Cao^1^*, Mingyang Chen^1^**

*^1^Beijing Computational Science Research Center, Beijing 100193, China*

*^2^School of Materials Science and Engineering, Sun Yat-sen University, Guangzhou 510275, P.R. China*

*^3^* *School of Material Science and Engineering, Hebei University of Technology, Tianjin, 300130, PR China*

Email: Mingyang Chen: [mychen@csrc.ac.cn](mailto:mychen@csrc.ac.cn)

Tengfei Cao: Tengfei.Cao@ csi.cuny.edu

Xueguang Chen: xgc@hebut.edu.cn


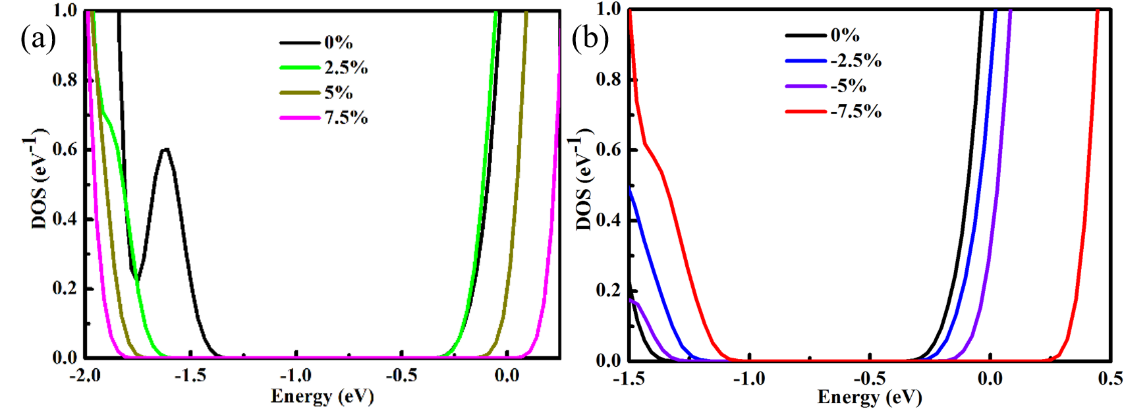


**Figure S1.** The enlarged drawing of aligned TDOS for the CH_3_NH_3_PbI_3_ systems under tensile strains (a) and compression strains (b). I core s band is used as the reference for the alignment.


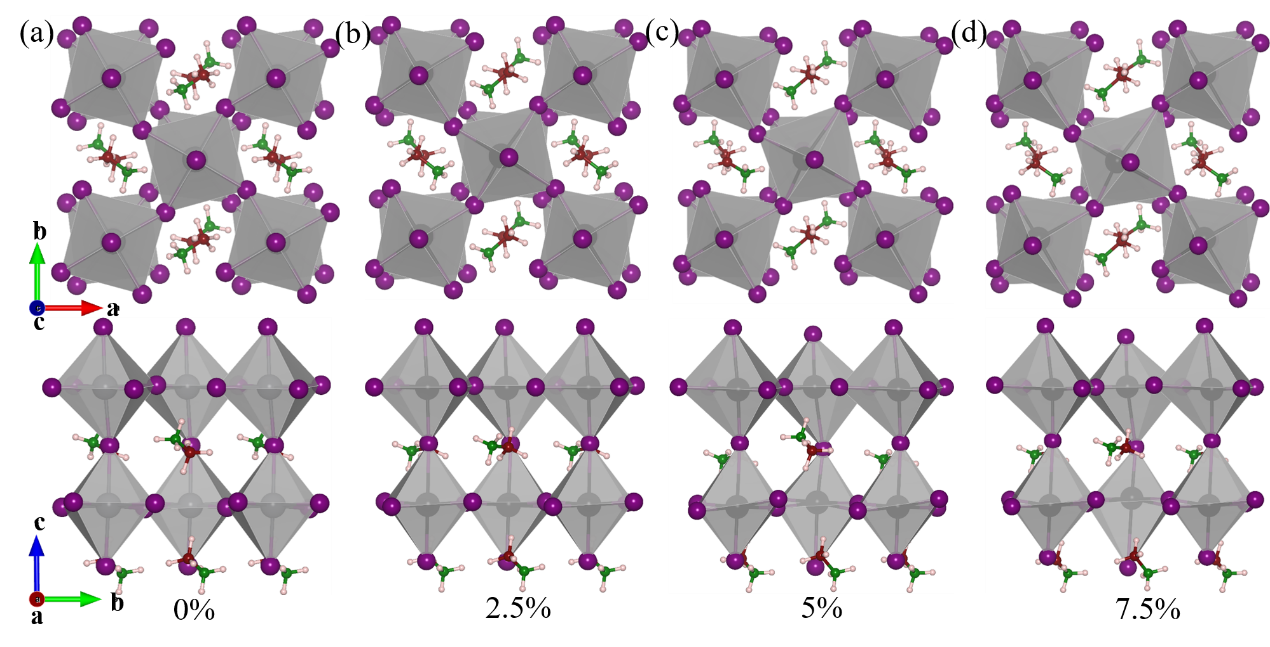


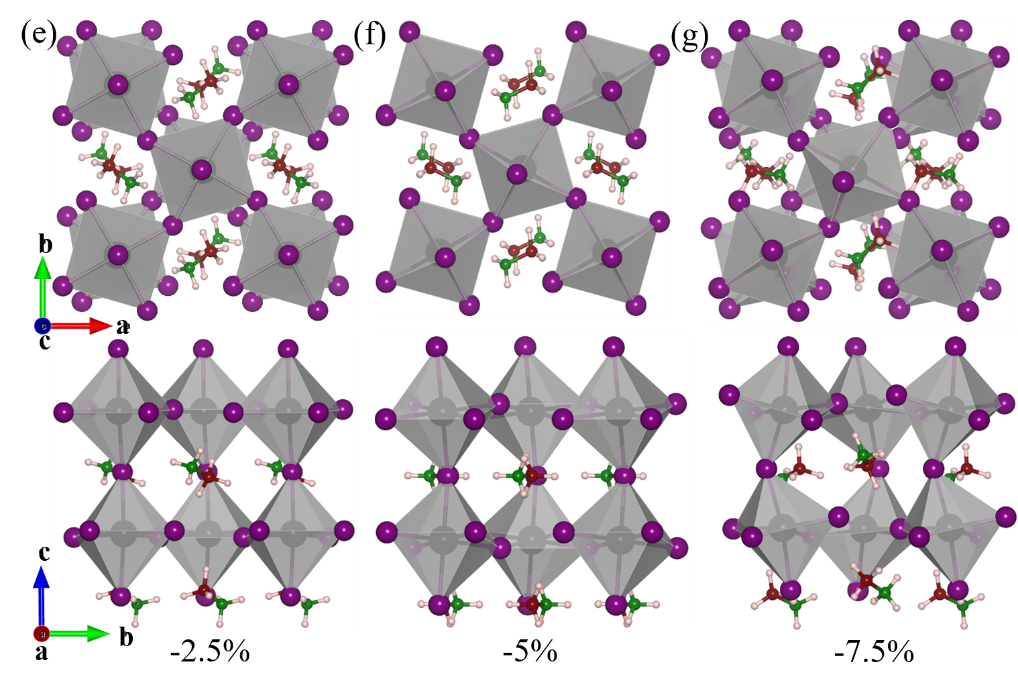


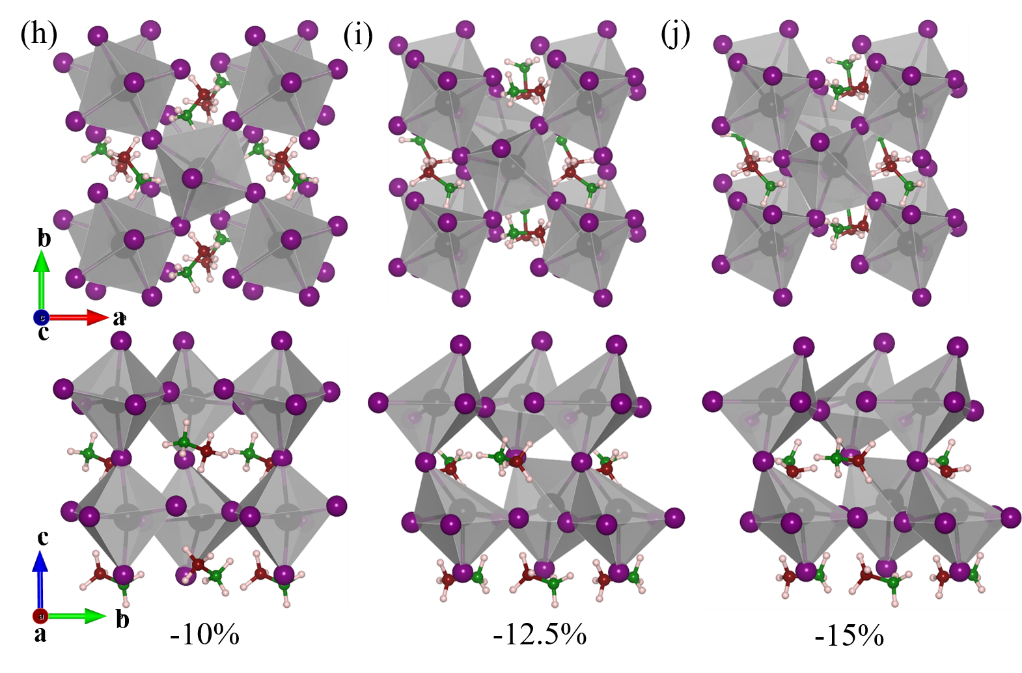


**Figure S2.** The top and side views of polyhedral structures of CH_3_NH_3_PbI_3_ under various strains: 0% (a), 2.5% (b), 5% (c), 7.5% (d), -2.5% (e), -5% (f), -7.5% (g), -10% (h), -12.5% (i), -15% (j).

**Table S1.** The lattice constants of CH_3_NH_3_PbI_3_ with different strains and the corresponding pressures (KB).

| Strain (%) | | 7.5 | 5 | 2.5 | 0 | -2.5 | -5 | -7.5 | -10 | -12.5 | -15 |
| --- | --- | --- | --- | --- | --- | --- | --- | --- | --- | --- | --- |
| Lattice constants (Å) | a | 9.61 | 9.39 | 9.16 | 8.94 | 8.72 | 8.49 | 8.27 | 8.05 | 7.82 | 7.60 |
|  | b | 9.61 | 9.39 | 9.16 | 8.94 | 8.72 | 8.49 | 8.27 | 8.05 | 7.82 | 7.60 |
|  | c | 13.95 | 13.63 | 13.30 | 12.98 | 12.66 | 12.33 | 12.01 | 11.68 | 11.36 | 11.03 |
| Pressure (KB) | | -13.65 | -11.61 | -10.67 | 0 | 14.90 | 30.65 | 53.97 | 66.70 | 119.23 | 172.78 |
